# A French Translation of the Pleasure Arousal Dominance (PAD) Semantic Differential Scale for the Measure of Affect and Drive

**DOI:** 10.5334/pb.340

**Published:** 2017-03-13

**Authors:** Sandrine Detandt, Christophe Leys, Ariane Bazan

**Affiliations:** 1 Service de Psychologie Clinique et Différentielle, Centre de Recherche en Psychologie Clinique, Psychopathologie et Psychosomatique, Université Libre de Bruxelles (ULB), Avenue Roosevelt 50 - CP 122, B-1050 Bruxelles, BE; 2 Unité de Recherche de Psychologie Sociale, Université Libre de Bruxelles (ULB), Avenue Roosevelt 50 - CP 122, B-1050 Bruxelles, BE

**Keywords:** Pleasure, Arousal, Dominance, Semantic Differential, French validation

## Abstract

Multivariate studies have repeatedly confirmed that three basic dimensions of human emotional behavior, called *pleasure* (P), *arousal* (A) and *dominance* (D) are persistent in organizing human judgments for a wide range of perceptual and symbolic stimuli. The Mehrabian and Russell’s PAD semantic differential scale is a well-established tool to measure these categories, but no standardized French translation is available for research. The aim of this study was to validate a French version of the PAD. For this purpose, (1) Mehrabian and Russell’s PAD was translated through a process of translations and back-translations and (2) this French PAD was tested in a population of 111 French-speaking adults on 21 images of the International Affective Picture System (IAPS). A confirmatory factor analysis revealed the expected three-factor structure; the French PAD also distributed the images in the affective space according to the expected boomerang-shape. The present version of PAD is thus a valid French translation of Mehrabian and Russell’s original PAD.

## General Introduction

Many researchers (Dickinson & Dearing, 1979; Hebb, 1955; Konorski, 1967; Lang, 2010; Mehrabian & Russell, 1974) have shown that survival strategies are the core mechanism for physiological mobilization and action. Specifically, this survival “law of effect” (Thorndike, 1898) predicts that when a species is offered a choice between two alternatives, it will approach elements with satisfying (so-called hedonic) and life-sustainable consequences and fight or flight alternatives with undesirable consequences (defensive motivation), and that it will repeat this behaviour in time (even if some results have not confirmed this pattern, see e.g., Mitchell, Osborne, & Boyle, 1985). Obviously, these survival strategies that guide human behaviour are more difficult to understand, because they are also affected by many other factors, including personal, situational and cultural imperatives. Wundt (1896) nevertheless proposed that three main dimensions underlie human behavior, which he labeled *Lust* (pleasure), *Spannung* (tension), and *Beruhigung* (inhibition).

Wundt’s theoretical categories have been repeatedly confirmed by empirical work, which has shown that these categories are persistent in organizing human judgments for a wide range of perceptual and symbolic stimuli. One such empirical contribution is the extensive research of Osgood and colleagues (1957) using semantic differential (or SD) scales. The SD scales measure people’s reactions to various stimuli and concepts in terms of ratings on bipolar scales defined with contrasting adjectives at each end (Heise, 1970). They found that, despite cultural linguistic differences, three dimensions of affective meaning were universal across cultures, namely *evaluation* (example of an item: “good-bad”), *activity* (example of an item “active-passive”) and *potency* (example of an item: “powerful-weak”). In 1974, following Osgood, Mehrabian and Russell also proposed three basic independent categories of affective meaning, which they called respectively *pleasure, arousal* and *dominance* (or PAD), and which transcend the sensory dimensions of judging. Using a modified and expanded version of the SD, they were able to demonstrate support for the PAD categories. Mehrabian and Russell (1974) conceived *pleasure* as a feeling that runs along a continuum ranging from unhappiness to extreme happiness, which they evaluated with word pairs like “pleased-annoyed” or “happy-unhappy”. They considered that it should be easily assessed (e.g., by self-reported ratings on SD scales, or by behavioral indicators such as smiles, laughter etc.). Mehrabian and Russell defined *arousal* as a mental activity that can be described along a single dimension ranging from sleep to excitement and linked to adjectives such as “stimulated-relaxed” and “excited-calm”. *Dominance* was described as being related to feelings of control along a continuum from dominance to submissive, with adjectives like “controlling-controlled” and “important-awed” (Bakker, Van der Voordt, Vink, & de Boon, 2014) Although Osgood’s categories and Mehrabian and Russell’s categories bear some similarities, the way they are defined show dissimilarities as well. For example, Osgood’s dimensions concern the judgment of the environment, and as such pertain to external stimuli, whereas Mehrabian and Russell describe the observer’s internal states (for a review, see Bakker et al., 2014). Nowadays, even if different interpretations of the concepts coexist, the Mehrabian and Russell’s PAD scale is the semantic scale that is predominantly used and which is still considered to be valid (Bakker et al., 2014).

Based on these different studies, Bradley and Lang (1994) designed the Self-Assessment Manikin Scale (or SAM), which is a non-verbal scale that uses humanoid figures to depict gradations along the same three evaluative dimensions: (1) *valence* (low = “unhappy/unsatisfied”; high = “happy/pleased”), (2) *arousal* (low = “calm, relaxed”; high = “excited, aroused”), and (3) *dominance* (low = “submissive, controlled”; high = “dominant, in control”). This tool is called the Self-Assessment Manikin Scale (or SAM). Bradley and Lang (1994) used the SAM, together with the PAD and asked participants to rate their feelings on 21 images of the International affective Picture System (or IAPS). This database consists of 1182 pictures representing different aspects of life and acting as potent elicitors of emotions (Lang, Bradley, & Cuthbert, 2008). These images have been used in many laboratories and in numerous types of protocols (e.g., Drace, Efendic, Kusturica, & Landžo, 2013; Lohani, Gupta, & Srinivasan, 2013; Verschuere, Crombez, & Koster, 2001).

Bradley and Lang (1994) found that, as expected, the corresponding categories of the PAD and the SAM were strongly correlated well and moreover, resulted both, independently, in affective spaces with the same pattern, a so-called “boomerang” pattern. This pattern showed that the more affectively connoted a stimulus was (positively or negatively), the more it was arousing. It should also be noted that the third dimension, dominance, has been repeatedly shown to account for much less variance than the two first dimensions (typically less than 15%).

As we can see, these different research lines converge to the observation that, despite the plethora of terms people use to describe their feelings, the underlying structure of affective language seems to have a relatively simple dual structure of valence and arousal (Lang, 2010). According to Bradley and her colleagues (2001), this structure encourages the hypothesis of a more general, underlying, biological determination. Indeed, and by way of simplification, we could say that valence is an affective component and arousal a drive component, and that affect and drive effectively capture the complexity of human behaviour.

However, although researchers seem to generally agree on this two-factor structure of human behaviour, the issue of how to assess it is much more complex. Basically, there are three ways to assess an individual response to a stimulus: by auto- or hetero- (verbal) reports, by physiological reactivity or by behavioral responses. In any case, an added difficulty is to choose among the large number of tests and paradigms. SAM is currently the more commonly used instrument, as it is a non-verbal and quick way to measure these dimensions (Morris, 1995). Although SAM is widely used in psychology research on emotions, it has only a single item-measure (i.e., one Manikin range) for each factor, which limits its possible use when compared to other instruments that can be subject to factor analysis. Therefore, we consider that the PAD has some advantage over the SAM in assessing emotions.

The aim of the present research is to validate a French translation of the PAD (‘French PAD’). Until now, there was no such translation, and strikingly, researchers limited themselves to selectively use only a few items from the original PAD, which were translated in an *ad hoc* non-validated way (e.g., Lehu, 2002; Lemoine, 2012). Therefore, the present paper first proposes a French translation of the original PAD by applying a combination of translations by native speakers, back-translations and expert reviews, which is the most rigorous way to validate the translation of an assessment tool (Brislin, Lonner, & Thorndike, 1973; Cha, Kim, & Erlen, 2007; Desrichard, Vos, Bouvard, Dantzer, & Paignon, 2008; Im, Page, Lin, Tsai, & Cheng, 2004; Tansuhaj & Foxman, 1996). Second, this French version will be tested on a population of French speaking adults for the 21 images of the IAPS used in Bradley and Lang’s (1994) study. Validation of our translation will be based upon two types of analyses: (1) the three-factor structure of the responses on the French PAD will be verified by factor analysis; (2) the predictive validity of our measure will be investigated by examining if the affective space derived from the French PAD follows a boomerang shape similar to the one found by Bradley and Lang (for the SAM and the PAD respectively).

## Translation Procedure

### Instrument

The PAD is composed of three different subscales (1) *pleasure* (2) *arousal* and (3) *dominance,* consisting of six items each. These items are all bipolar pairs of adjectives, at the outer poles of a nine-point Likert scale. The rating scale thus ranged from –4 to +4, with 0 representing the center segment of the scale. Specifically, Mehrabian and Russell (1974) assess *pleasure* in terms of respondents’ verbal assessment of their reactions to the environment as: “happy” (as opposed to “unhappy”); “pleased” (“annoyed”); “satisfied” (“unsatisfied”); “contented” (“melancholic”); “hopeful” (“despairing”) and “relaxed” (“bored”). *Arousal* is assessed by verbal reactions to an environment as: “stimulated” (as opposed to “relaxed”); “excited” (“calm”); “frenzied” (“sluggish”); “jittery” (“dull”); wide-awake (“sleepy”) and “aroused” (“unaroused”). Finally, *dominance* is reflected in verbal appraisals in which the respondent feels: “controlling” (as opposed to “controlled”); “influential” (“influenced”); “in control” (“cared-for”); “important” (“awed”); “dominant” (“submissive”) and “autonomous” (“guided”). (See also Table 1, first column).

**Table 1:** Summary of the translations of the items of Russell and Mehrabian’s (1974) semantic differential (column 1); items are polarized adjective pairs for the dimensions *pleasure, arousal* and *dominance*. The blue items are the ones kept in the final French PAD (column 2). The final items of the French PAD appear at least once in one of the two expert translations (columns 3 and 4). The * reminds the reader the original pair “relaxed-bored”.

ORIGINAL PAD	FRENCH PAD	TRANSLATOR I	TRANSLATOR II
(English instrument)	(Final translation)		

**Happy-Unhappy**	Heureux(se)-Malheureux(se)	Satisfait(e)-Malheureux(se)	Heureux(-se)-Malheureux(se)
**Pleased-Annoyed**	Content(e)-Contrarié(e)	Content(e)-Contrarié(e)	Content(e)-Mécontent (e)
**Satisfied-Unsatisfied**	Satisfait(e)-Insatisfait(e)	Satisfait(e)-Insatisfait(e)	Satisfait(e)-Insatisfait(e)
**Contented-Melancholic**	Epanoui(e)-Mélancolique	Heureux(se)-Mélancolique	Epanoui(e)-Mélancolique
**Hopeful-Despairing**	Confiant(e)-Désespéré(e)	Confiant(e)-Désespérée	Confiant(e)-Désespéré(e)
Amused*-Bored	Amusé(e)-Ennuyé(e)	Amusé(e)-Ennuyé(e)	Détendu(e)-Ennuyé(e)
**Stimulated-Relaxed**	Stimulé(e)-Détendu(e)	Stimulé(e)-Détendu(e)	Stimulé(e)-Décontracté(e)
**Excited-Calm**	Excité(e)-Calme	Excité(e)-Calme	Excité(e)-Calme
**Frenzied-Sluggish**	Frénétique-Léthargique	Exalté(e)-Léthargique	Frénétique-Léthargique
**Jittery-Dull**	Nerveux(se)-Mou(lle)	Nerveux(-se)-Morne	Nerveux(-se)-Mou(lle)
**Wide awake-Sleepy**	Bien réveillé(e)-Somnolent(e)	Actif(-ve)-Somnolent(e)	Bien réveillé(e)-Endormi(e)
**Aroused-Unaroused**	Animé(e)-Amorphe	Animé(e)-Non stimulé(e)	Eveillé(e)-Non stimulé(e)
**Controlling-Controlled**	Contrôlant(e)-Etre contrôlé(e)	Contrôlant(e)-Etre contrôlé(e)	Contrôlant(e)-Etre contrôlé(e)
**Influential-Influenced**	Influent(e)-Influencé(e)	Influent(e)-Influencé(e)	Influent(e)-Influencé(e)
**In control-Cared for**	Maître de soi-Pris en charge	Maître de soi-Sous la charge	Aux commandes-Pris en charge
**Important-Awed**	Important(e)-Impressionné(e)	Important(e)-Impressionné(e)	Important(e)-Impressionné(e)
**Dominant-Submissive**	Dominant(e)-Soumis(e)	Dominant(e)-Soumis(e)	Dominant(e)-Soumis(e)
**Autonomous-Guided**	Autonome-Guidé(e)	Autonome-Guidé(e)	Indépendant(e)-Guidé(e)

### Translation

Two native French speakers who were expert in English translated each word pair independently. The process first privileged literal translation (for example: satisfied-*satisfait*) as well as the fact that the adjectives were paired by two and had to be opposed. We asked the translators to take this dichotomy into account. This consideration led us to modify the original English pair “relaxed-bored”, which actually does not constitute a semantic opposition – as “relaxed” is not the antonym of “bored”. As such, in its original version, the translators could not agree over the translation of this word pair. Moreover, the word “relaxed” appears twice in the original version: once in the *pleasure* dimension with “relaxed-bored” and once in the *arousal* dimension with “relaxed-stimulated”. For all these reasons we decided to change this pair in the original English version from “relaxed-bored” to “amused-bored” and proposed this pair for final translation. This allowed a unambiguous translation/back-translation and corresponded better to with the theory that this dimension spreads along a continuum of pleasure-displeasure. As a result, out of the 72 adjectives, 57 (79 per cent) were identical between the two translations, which means a high degree of agreement between the two translators. The results of both translators are given in Table 1. In the final version, each adjective was always given by at least one of the two translators, except for one item. For the pairs for which we obtained two different translations, we requested back-translation and we kept the French adjective which corresponded with the original English version. For the pair “aroused-unaroused” for which we chose another translation, both translators had “unaroused” translated as “non stimulé”, which is not a French adjective but rather a negation of a French word; we therefore replaced the translation with the French word “amorphe” (“lifeless” in English). Eventually, the final translation (see column 2 of Table 1) went through back-translation.

### Back-translation

Two new experts, English native speakers, separately performed a back-translation. This stage was necessary as we were not so much concerned by a literal translation but with generating a meaning that was as similar as possible to the original English version. In other words, the objective was to ensure the cross linguistic equivalence of the instrument.

The results of the two independent back-translations (see Table 2 below) showed that out of the 72 adjectives, 52 (72 per cent) were identical, which means a good degree of agreement between the two translators. When comparing the two back-translations with the English instrument, there were 12 differences. From these, seven items gave only very slight differences when retranslated to English and kept essentially the same meaning; the item “insatisfait” was translated as “displeased” instead of “unsatisfied” by one translator; item “désespéré” as “hopeless” instead of “despairing” and item “somnolent” as “sleepy” instead of “unawakened”. For the five remaining pairs, three pairs and one word were never reproduced by any translator: “frénétique-léthargique” was back-translated as “exhalted-lethargic”, while the original version was “frenzied-sluggish”; “nerveux-mou” was back-translated as “nervous-sad” while the original version was “jittery-dull”; “animé-amorphe” was back-translated as “awake-under stimulated” while the original version was “aroused-unaroused” and the word “contrarié” was back-translated as “upset” while the original version was “annoyed”. However, back-translations rarely lead to perfect matches with the original version (Cha et al., 2007) and we considered this result satisfactory for further validation of the instrument. Therefore, after two translations and two back-translations, both with a high degree of agreement, the French version of the instrument was administered to a sample of Belgian French-speakers for empirical validation.

**Table 2:** Summary of the back-translations of the final French PAD (column 2). The blue items are the ones that correspond to the original Russell and Mehrabian’s (1974) semantic differential (column 1) (column two). 72 adjectives, 60 (83 per cent) of the two back-translations (columns 3 and 4) were identical to the original version.

ORIGINAL PAD	FRENCH PAD	TRANSLATOR I	TRANSLATOR II
(English instrument)	(Final translation)		

**Happy-Unhappy**	Heureux(-se)-Malheureux(-se)	Happy-Unhappy	Happy-Unhappy
**Pleased-Annoyed**	Content(e)-Contrarié(e)	Pleased-Upset	Pleased-Upset
**Satisfied-Unsatisfied**	Satisfait(e)-Insatisfait(e)	Satisfied-Displeased	Satisfied-Unsatisfied
**Contented-Melancholic**	Epanoui(e)-Mélancolique	Content(ed)-Melancholic	Content(ed)-Melancholic
**Hopeful-Despairing**	Confiant(e)-Désespéré(e)	Hopeful-Hopeless	Optimistic-Despairing
**Relaxed-Bored**	Amusé(e)-Ennuyé(e)	Relaxed-Bored	Relaxed-Bored
**Stimulated-Relaxed**	Stimulé(e)-Détendu(e)	Stimulated-Mellow	Stimulated-Relaxed
**Excited-Calm**	Excité(e)-Calme	Excited-Calm	Excited-Calm
**Frenzied-Sluggish**	Frénétique-Léthargique	Exhalted-Lethargic	Exhalted-Lethargic
**Jittery-Dull**	Nerveux(-se)-Mou	Nervous-Sad	Nervous-Sad
**Wide awake-Sleepy**	Bien réveillé(e)-Somnolent(e)	Awake-Sleepy	Awakened-Unawakened
**Aroused-Unaroused**	Animé(e)-Amorphe	Awake-Under stimulated	Awake-Under-stimulated
**Controlling-Controlled**	Contrôlant(e)-Etre contrôlé(e)	Controlling-Controlled	Controlling-Controlled
**Influential-Influenced**	Influente)-Influencé(e)	Influential-Influenced	Influential-Influenced
**In control-Cared for**	Maître de soi-Pris en charge	In control-Cared for	In control-Under the authority of
**Important-Awed**	Important(e)-Impressionné(e)	Important-Awed	Important-Awed
**Dominant-Submissive**	Dominant(e)-Soumis(e)	Dominant-Submissive	Dominant-Submissive
**Autonomous-Guided**	Autonome-Guidé(e)	Autonomous-Guided	Autonomous-Guided

## Validation Study

We tested the validity of the French PAD through the replication of the Bradley and Lang (1994) study in order to verify 1) its factorial structure and 2) the distribution of the evaluation of the IAPS images in the affective space using the French PAD.

### Participants

The sample consisted of 111 participants (26 men) vs. 78 participants in the original Bradley and Lang (1994) study and the mean age was 31.38 (SD: 13.07; range 18–65). Participants were recruited via email, through personal contacts and by announcements on/advertising through social networks (the research goal was described and a link to the survey was provided). Participants completed the online survey anonymously, each on their personal computer. The survey (Limesurvey Version 2.05+ Build 150413) included requests for demographic information and the presentation of 21 IAPS images, which people had to rate on the PAD.

Even if the normative rating procedure used by Bradley and Lang (1994) was followed as closely as possible, the data quality of an online survey can be of some concern. Therefore, we followed Meade and Craig’s (2012) recommendations. First, people were required to partially identify themselves (if they were students, which was the case for 28 of them, they had to give their student registration number and if not, they could give their email to receive feedback about the study) and second, we added 3 bogus questions (e.g., “Respond with ‘strongly agree’ for this item”) randomly throughout the test. All participants answered these questions correctly and only one gave his email to receive feedback. Results did not differ regarding the origin of recruitment (university or social network).

### Procedure

On the first page of the survey, participants were told that they would see different pictures representing different events and that they would have to rate the reaction that these pictures evoked on different scales. We warned them that some images could be disturbing.

After that, they gave informed consent, indicate their language proficiency level and age. If they were not fluent French speakers or if they were under 18, they were unable to continue with the test. After reading the standardized instructions (Mehrabian & Russell, 1974, Appendix B),^1^ the experiment started. They were presented one of the images for six seconds; the presentation order was randomised over the participants. Next, the browser refreshed the screen to a new page made of the 18 dichotomous pairs of words (six pairs for each dimension). Each pair was presented the same way (negative valence, unaroused and dominated items on the left side) but the pairs themselves were randomly presented. Participants had to rate their reaction to each image on a nine-point Likert scale for the 18 items of the French PAD. A general debriefing was sent by email, and people were invited to ask for further information if they wished.

### Images

Twenty-one pictures that varied in pleasantness were selected from the International Affective Picture System (Lang, Bradley, & Cuthbert, 2008) and were the same as the ones used in Bradley and Lang (1994). The IAPS pictures used were: 1090, 1240, 1500, 2040, 2110, 2200, 2500, 3010, 3150, 4610, 5000, 5600, 6230, 7000, 7270, 8030, 9090, 9140, 9160, and 2 erotic pictures, 4180 (a picture of a naked woman) and 4520 (a picture of a naked man). These pictures depicted objects such as a snake, spider, gun, mutilated face, rolling pin, soldier, flowers, mountains, cake, baby, and others. In contrast to Bradley and Lang (1994), who presented two erotic female pictures to men and two erotic male pictures to women, we presented the same erotic pictures (one female, one male) for both men and women. Therefore, we left out picture 4220 (picture of a naked woman) for the men and 4500 (picture of a naked man) for the women, in order to keep the number of pictures presented equal for both men and women and the number of pictures equal with the number in the original series (see the table of descriptive statistics as supplemental material showing, for each image, the mean, skewness and kurtosis of each factor rating).

## Results

### Summary of data

**Figure 1:**
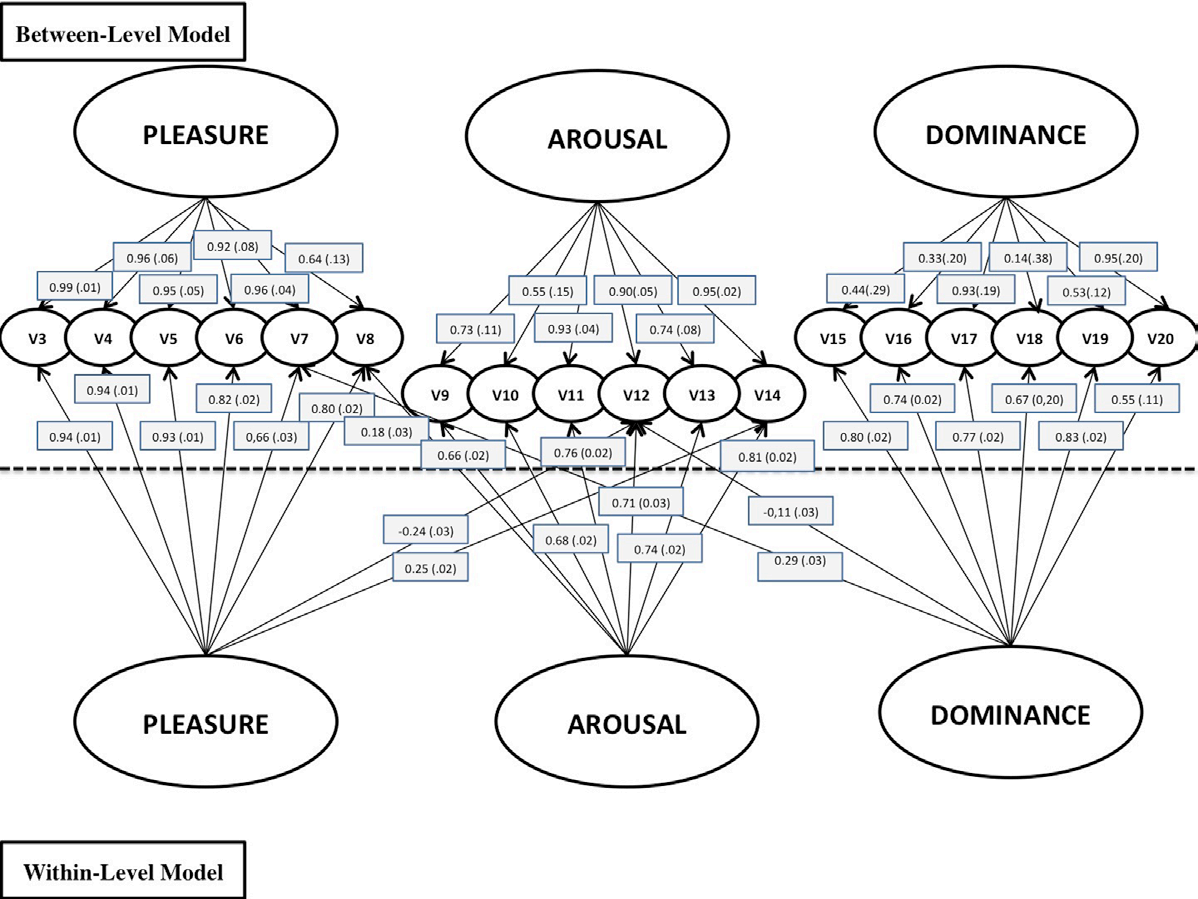
Multilevel model re-specified at the within level. For the sake of clarity, the disturbance of the variables is not represented but a correlation disturbance between v9 and v10 has been estimated. Selected estimates values for the within (images) level and SE in parenthesis.

Because we used a within-subjects design (all subjects had to rate each image on the three factors), we can not assume that the errors are independent. Indeed, intraclass correlations (ICC, McGraw & Wong, 1996) were always above .1 and up to .56 (see Table 3 and 4), therefore a multilevel confirmatory factor analysis was computed (Byrne, 2010). This analysis showed that dependency problems in the model required the following modifications. First, five cross-loadings had to be estimated: v12 (Nerveux(-se)-Mou/lle) and v14 (Animé(e)-Amorphe) also loading on factor one (pleasure) in the between level; v8 (Amusé(e)-Ennuyé(e)) also loading on the factor two (arousal); v7 (Confiant(e)-Désespéré(e)) and v12 also loading on the factor three (dominance). Second, the perturbations of v9 (Stimulé(e)-Détendu(e)) and v10 (Excité(e)-Calme) were strongly correlated, hence we had to estimate that correlation (instead of assuming a zero value). Taking these cross-loadings and correlation perturbation into account (see Figure 1), we obtain adequate model fit in terms of the CFI, GFI and the RMSEA values (CFI = .95; GFI = .94; RMSEA = .037). Although the chi-square (χ(258)^2^ = 1065.2) was significant (p < .001), it is important to note that, given the large number of degrees of freedom, the test has a very high statistical power and detects any departure from the theoretical distribution. To avoid this sensitivity bias, another fit index can be computed by dividing the chi-square by the number of degrees of freedom (chi-square/df = 4.13, p < .001). This new chi-square value, then, is considered as acceptable since if it is below the cut off of 5 (Byrne, 2010; Fossati, Somma, Karyadi, Cyders, & Borroni, 2015).

**Table 3:** Intraclass correlations (or ICC, i.e., the proportion of between-group variance in the total variance) for each of the observed variables (or items) of the PAD; *N* = 111.

Variable	ICC	Variable	ICC	Variable	ICC

**V3**	0.04	V4	0.02	V5	.03
**V6**	0.06	V7	0.07	V8	.05
**V9**	0.13	V10	0.13	V11	.15
**V12**	0.09	V13	0.32	V14	.15
**V15**	0.05	V16	0.56	V17	.20
**V18**	0.02	V19	0.04	V20	.11

**Table 4:** Abbreviated PAD scale Item Content of all 18 variables which represent a pair of the PAD scale (numbered from V3 to V20) and the two levels of analysis (V1: participants, V2 images).

Variables	Content	Variables	Content

**V1**	Participant	**V11**	Frénétique-Léthargique
**V2**	Image	**V12**	Nerveux(-se)-Mou/lle
**V3**	Heureux(-se)-Malheureux(-se)	**V13**	Bien réveillé(e)-Somnolent(e)
**V4**	Content(e)-Contrarié(e)	**V14**	Animé(e)-Amorphe
**V5**	Satisfait(e)-Insatisfait(e)	**V15**	Contrôlant(e)-Etre contrôlé(e)
**V6**	Epanoui(e)-Mélancolique	**V16**	Influent(e)-Influencé(e)
**V7**	Confiant(e)-Désespéré(e)	**V17**	Maître de soi-Pris en charge
**V8**	Amusé(e)-Ennuyé(e)	**V18**	Important(e)-Impressionné(e)
**V9**	Stimulé(e)-Détendu(e)	**V19**	Dominant(e)-Soumis(e)
**V10**	Excité(e)-Calme	**V20**	Autonome-Guidé(e)

Therefore, we conclude that the French PAD shows the same three-factor structure (*pleasure, arousal* and *dominance*) as the original PAD.

### Reliability of the scale

Although the internal consistency of the three factors of Mehrabian and Russell’s PAD is well established, for the French PAD it was necessary to verify the unidimensionality of the three scales. Results on the Cronbach’s alpha for the different images indicated a good internal consistency with scores ranging from .84 to .95 for the *pleasure* scale with a mean of .91; from .75 to .97 for the *arousal* scale with a mean of .85 and from .71 to .92 for the *dominance* scale with a mean of .84. These results corroborate the validity of the French PAD.

### The affective space

**Figure 2:**
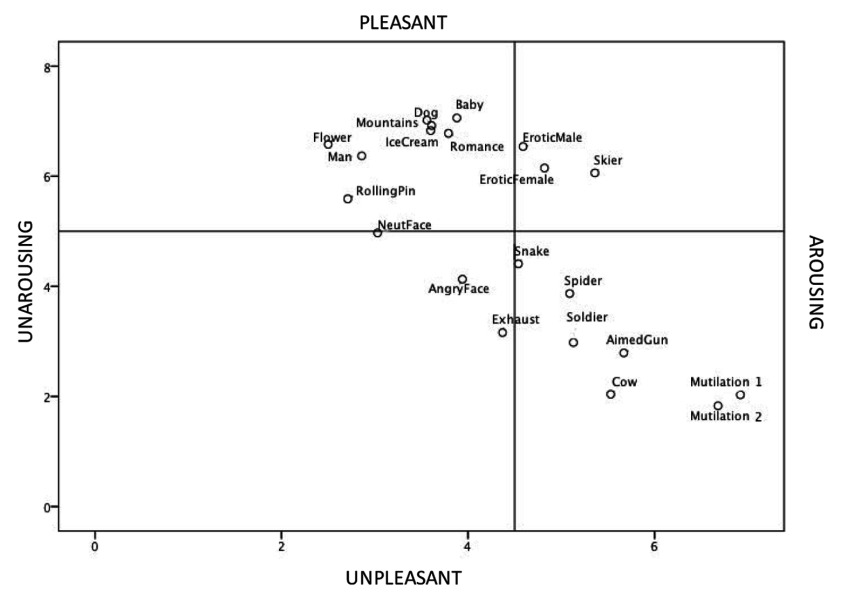
Distribution of 21 IAPS-pictures in a 2-dimensional affective space defined by the French PAD valence and arousal ratings.

The mean variance component explained by the *pleasure* dimension on the different images is 41.3% (ranging from 33.6 to 50.4), by the *arousal* dimension 15.7% (ranging from 8.4 to 21.3) and by the *dominance* dimension 8.9% (ranging from 5.9 to 11.04). As the *dominance* dimension explains less of the variance in affective ratings, and in accordance with past practice (e.g., Bradley & Lang, 1994; Drace, et al., 2013; Lohani, et al., 2013; Verschuere, et al., 2001), we plotted the affective space represented by our French PAD exclusively as a function of the *pleasure* and on the *arousal* scales. As can be seen in Figure 2, the 21 IAPS-pictures are plotted in a two-dimensional affective space, given by the poles pleasant-unpleasant for *pleasure*, and arousing-unarousing for *arousal*. The shape of the affective space shows a boomerang pattern: clearly, *pleasure* and *arousal* are not linearly correlated, but both increases in either pleasure or displeasure tend to correlate with increases in arousal. To show this effect, we computed a regression analysis with valence set as DV and arousal linear and quadratic set as IVs and it was significant, F(2, 2307) = 13.03, p < .001. It yielded both a linear (β = .38, p < .001) and quadratic (β = –45, p < .001) effect.

## Discussion

The PAD is a tool that is now largely used in economic psychology and is no longer applied to the psychology of emotions (Bradley & Lang, 1994). However, we think it remains an interesting tool for psychology in general, and for emotion psychology in particular, as it proposes a fine-grained way to assess an emotional state distinguishing *pleasure, arousal* and *dominance*. In order to reduce the variability due to local and non-standardized translations and to allow this measure to be applied to a wider domain of application than the one in which it is currently used, the present study proposes the validation of a French PAD. First, we translated the original versions by ways of translations and back-translations, and second, we applied this French PAD on standardized stimuli (namely, IAPS images) according to the procedures of the original Bradley and Lang (1994) study, using a sample of 111 participants.

Through a process of translation, back-translation and expert review, we obtained a stable French translation of the original PAD with high agreement between translators. As the original English version is already validated, this allowed us to conduct a CFA in order to test whether the theoretical tripartite division of the original version of the PAD also applied to our French version. This analysis appears to yield valid measures for the factorial structure of the tool as shown by the CFA. The boomerang-shaped affective space and the good internal consistency revealed by the Cronbach’s alphas corroborate previous studies as well. In order to validate the three-factor structure of our instrument, we had to tackle a number of statistical issues, which obscured the original Mehrabian and Russell (1974) and Bradley and Lang (1994) validations. Bradley and Lang (1994), for example, analyzed a hypothetical three-factor structure on the basis of PAD scores by averaging the scores for all the images for each participant. For example, image 2040 (baby) will generally be assessed as pleasant and not arousing, but image 3150 (bloody hand) should elicit the opposite pattern (i.e., unpleasant and arousing) while image 8030 (skier) induces both a pleasant and an arousing feeling. Therefore, even if the factorial structure exists, it cannot be correctly detected with single-level analysis through averaging the scores on all the images for each participant because this would aggregate all the variability due to the nature of the images within this organizational level. Another important problem is that the exploratory factor analysis used by Mehrabian and Russell and Bradley and Lang violated a major statistical assumption, namely that all observations are independent. However, this is obviously not the case as the evaluation from one participant for one image is partially dependent on the evaluation the same participant’s evaluation of another image (Byrne, 2011). We avoided this bias by using the MLV CFA. Indeed, in contrast to single-level analysis, multilevel modeling allows us to consider both levels of the hierarchically structured data simultaneously, i.e., both participant- and image-related variability.

The present findings corroborate the observation that the dimensions of *pleasure, arousal* and *dominance* are capable of representing an individual’s feelings over a variety of contexts, as has been previously shown by numerous authors using a wide variety of methods (Dickinson & Dearing, 1979; Hebb, 1955; Konorski, 1967; Lang, 2010; Mehrabian & Russell, 1974). The persistence of these findings over researchers, culture and time, seems to indicate that pleasure, and arousal – and possibly dominance to a lesser extent – form two (or three) basic dimensions of experience related to most aspects of human behaviour. This may be important for future research in many domains of psychology, as well as for interdisciplinary studies in between these psychological domains. For example, in some of our other work concerning addiction, we use the PAD in order to assess if the theoretical and clinical distinction (e.g., Robinson & Berridge, 2001) between *wanting* and *liking* has experimental support (see Bazan & Detandt, 2013, for a theoretical discussion). *Wanting* can be defined as the amount of energy an organism is ready to invest in order to obtain a reward and its neurobiological circuitry is thought to be located in the mesolimbic dopaminergic pathways. *Liking*, on the other hand, is what is expressed by facial and behavioural mimics conserved over species, such as smiles and laughter and its biological circuitry is thought to be situated in the subcortical opioid hedonic “hotspots”. Yet, as *valence* varies between positive or pleasant or appetitive and negative or aversive, and as *arousal* is defined as an intensity of bodily activation (Russell & Mehrabian, 1977), this dichotomy has remarkable parallels with the difference between *liking* and *wanting* respectively (see also Detandt, Askari, Olyff, & Bazan, 2014). In particular, addiction would be related to *wanting* without *liking*, as may be argued, to arousal without pleasure. As such, the PAD could be an effective way to reveal these relationships using addiction-related stimuli in a targeted population. Indeed, it would be of particular interest to examine if at some point in the course of addiction, such stimuli elicit more arousal in addicts as compared to controls, while not particularly inducing more pleasure. Such a prediction is suggested by the study of Moeller et al. (2012) with cocaine addicts showing a preference for cocaine images as compared to other stimuli independently of the associated pleasure level.

### Limitations and future directions

The Bradley and Lang (1994) study was a paper-pencil research with a student sample in the late 1990s while our study takes place 20 years later with an electronic version in a sample of both students and members of the general population. Moreover, it can be of some concern that the IAPS images do not elicit emotions in the same way or to the same extent as they did twenty years ago. These differences might bias the comparison between Bradley and Lang’s study and ours. However, as we find essentially comparable factor results, these differences do not seem to have interfered greatly. One limitation of our study is that we did not assess the test-retest reliability of the PAD, and thus we cannot be certain that the instrument yields stable results over time.

In order to further ensure the content and predictive validity of the French PAD, further research with the instrument should be done, for example, assessing time-related changes in affective reactions to contextual stimuli, as a function of therapy or other interventions. In particular, results of the French PAD could be compared with those on the SAM or the Emotion Reactivity Scale (Nock, Wedig, Holmberg, & Hooley, 2008) for the same stimuli. Taken together, our data indicate that our French translation of the PAD is a valid method for the measurement of emotional states and we hope it will be used in future research in combination with other indices of emotional responses.

## Additional File

The additional file for this article can be found as follows:

10.5334/pb.340.s1 Mean Valence, resp. Arousal and Dominance, skewness and kurtosis for the 21 images of the IAPS.

Mean Valence, resp. Arousal and Dominance, skewness and kurtosis for the 21 images of the IAPS. DOI: https://doi.org/10.5334/pb.340.s1

## Footnotes

Our translation: « Pour chaque image, nous allons vous demander de répondre à des paires d’affirmations/infirmations à propos de ce que vous viendrez de voir. Nous vous demandons d’y répondre en vous situant le plus proche de l’affirmation/infirmation qui décrit le mieux votre état par rapport à l’image que vous viendrez de voir. Certaines affirmations/infirmations pourront vous paraître inhabituelles mais vous vous sentirez probablement, malgré tout, plus proche d’une phrase que de l’autre. Parmi les différentes images, il y en aura pour lesquelles vous allez trouver certaines questions plus appropriées et d’autres moins. Merci d’essayer de répondre au mieux. »

## Concurrent submissions

There is no concurrent submission for this paper.
