# Supplementary material for: A French Translation of the Pleasure Arousal Dominance (PAD) Semantic Differential Scale for the Measure of Affect and Drive
Source: Psychol Belg. 2017 Mar 13;57(1):17–31. doi: 10.5334/pb.340 (PMC5808109; doi:10.5334/pb.340)
Supplement: Supplementary file 1 — Mean Valence, resp. Arousal and Dominance, skewness and kurtosis for the 21 images of the IAPS. [file pb-57-1-340-s1.pdf]

Number	Image	Valence			Arousal			Dominance		
		Mean (StD)	Skewness	Kurtosis	Mean (StD)	Skewness	Kurtosis	Mean (StD)	Skewness	Kurtosis
1090	Snake	4.82 ±1.1	0.82	1.92	5.93 ±1.2	-0.81	2.91	4.64 ±1.1	0.17	1.34
1240	Spider	4.76 ±1.4	0.24	-0.42	6.15 ±1.2	0.44	-0.19	4.84 ±1.4	0.21	0.31
1500	Dog	6.50 ±1.2	-0.09	-0.63	5.23 ±1.2	-0.13	1.51	5.78 ±1.1	1.00	0.45
2040	Baby	6.74 ±1.5	-0.56	0.29	5.58 ±1.1	-0.25	2.58	6.04 ±1.1	0.59	-0.34
2110	AngryFace	4.55 ±1.0	0.30	0.86	5.46 ±1.1	-0.24	1.23	4.83±1.1	-0.08	0.83
2200	NeutFace	5.03 ±1.2	0.51	1.11	4.86 ±1.2	-0.40	1.56	5.16 ±1.0	-0.06	1.84
2500	Man	6.10 ±1.3	0.26	-0.30	4 .79 ±1.1	-0.62	2.39	5.41 ±0.8	0.62	-1,12
3010	Mutilation1	3.12 ±1.4	0.14	-0.50	6.39 ±1.3	-0.01	-1.23	4.17 ±1.2	-0.59	0.56
3150	Mutilation2	3.45 ±1.3	0.11	-0.26	6.46 ±1.2	0.27	-0.39	4.13 ±1.2	-0.42	0.40
4180	EroticFemale	5.97±1.3	-0.14	0.72	5.93 ±1.5	-0.00	0.05	5.21 ±1.1	0.21	0.03
4520	EroticMale	6.35 ±1.3	-0.13	-0.31	5.74 ±1.4	-0.26	0.23	5.52 ±1.1	0.82	1.14
4610	Romance	6.26 ±1.7	-0.10	-0.71	5.15 ±1.4	0.39	0.37	5.59 ±1.2	0.86	1.20
5000	Flower	6.36 ±1.4	0.01	-0.10	4.50 ±1.2	-0.10	1.55	5.65 ±1.0	0.88	2.06
5600	Mountains	6.47 ±1.2	0.20	-0.79	5.12 ±1.3	-0.22	0.80	5.07 ±1.0	0.51	1.20
6230	AimedGun	3.68 ±1.3	-0.07	0.17	6.43 ±1.3	0.03	0.22	3.55 ±1.4	-0.19	-0.73
7000	RollingPin	5.59 ±1.4	0.62	0.31	4.89 ±1.4	-0.09	0.67	5.61 ±1.2	0.39	2.80
7270	IceCream	6.18 ±1.5	0.11	-0.46	5.42 ±1.4	-0.10	0.07	5.57 ±1.2	0.80	0.54
8030	Skier	6.00 ±1.3	0.57	0.09	6.23 ±1.5	-0,71	1.21	5.04 ±1.3	0.67	0.62
9090	Exhaust	3.58 ±1.4	0.26	0.38	5 .37 ±1.4	-0.23	1.22	4.73 ±1.2	0.26	1.87
9140	Cow	3.06 ±1.3	0.35	0.30	5.75 ±1.2	0.43	0.90	4.46 ±1.0	-0.75	0.70
9160	Soldier	3.44 ±1.3	0.04	-2.24	5.79 ±1.0	0.38	-1.12	4.33 ±1.2	-0.48	0.74

Supplemental table : Mean Valence, resp. Arousal and Dominance, skewness and kurtosis for the 21 images of the IAPS.
